# Considerations for the Consumption of Vitamin and Mineral Supplements in Athlete Populations

**DOI:** 10.1007/s40279-023-01875-4

**Published:** 2023-06-26

**Authors:** Peter Peeling, Marc Sim, Alannah K. A. McKay

**Affiliations:** 1 School of Human Sciences (Exercise and Sport Science), The University of Western Australia, Crawley, WA 6009 Australia; 2 Western Australian Institute of Sport, Mt Claremont, WA 6010 Australia; 3 Nutrition and Health Innovation Research Institute, School of Medical and Health Sciences, Edith Cowan University, Joondalup, WA 6067 Australia; 4 Medical School, The University of Western Australia, Crawley, WA 6009 Australia; 5 Mary MacKillop Institute for Health Research, Australian Catholic University, Melbourne, VIC 3000 Australia

## Abstract

Vitamins and minerals are of fundamental importance to numerous human functions that are essential to optimise athlete performance. Athletes incur a high turnover of key vitamins and minerals and are therefore dependent on sufficient energy intake to replenish nutrient stores. However, many athletes are poor at servicing their energy replenishment needs, especially female athletes, and although a ‘food first approach’ to meeting nutrient requirements is the primary goal, it may be important for some athletes to consider a vitamin and/or mineral supplement to meet their daily needs. When working to determine if an athlete requires vitamin or mineral supplements, practitioners should use a robust framework to assess the overall energy requirements, current dietary practices and the biological and clinical status of their athletes. Of note, any supplementation plan should account for the various factors that may impact the efficacy of the approach (e.g. athlete sex, the nutrient recommended dietary intake, supplement dose/timing, co-consumption of other foods and any food–drug interactions). Importantly, there are numerous vitamins and minerals of key importance to athletes, each having specific relevance to certain situations (e.g. iron and B vitamins are significant contributors to haematological adaptation, calcium and vitamin D are important to bone health and folate is important in the female athlete); therefore, the appropriate supplement for a given situation should be carefully considered and consumed with the goal to augment an athlete’s diet.

## Key Points

**Table:** 

Athletes are generally poor at servicing their energy intake requirements, and although a ‘food first approach’ is a primary goal, certain situations (i.e. food-restricted diets, poor recovery timing and nutrition, etc.) may warrant an athlete to consider a vitamin or mineral supplement to meet their daily needs.
There are numerous vitamins and minerals of key importance to athletes, each having specific relevance to certain situations. The usefulness of supplementation for a given situation should be carefully considered before use and consumed to augment an athlete’s typical diet.
Female athletes have nuanced vitamin and mineral requirements that differ to their male counterparts, and the research landscape requires significant work to better understand the unique nutritional challenges they face.

## Introduction

Vitamins and minerals play a fundamental role in a plethora of human processes that are of significance to athlete health and performance. Movement-dependent functions, such as energy metabolism, oxygen transport, red blood cell production, immune function, muscle growth/repair, and bone health, are all dependent on vitamins and minerals in some way. However, in most instances, the body is unable to endogenously synthesise these essential nutrients, and therefore, humans are dependent on exogenous food sources to supply our body’s vitamin and mineral needs. Of note, athletes present with an increased overall demand for vitamins and minerals, which is generally well-achieved when energy demands are met with appropriate levels of energy intake using a ‘food first approach’; defined as, ‘where practically possible, nutrient provision should come from whole foods and drinks rather than from isolated food components or dietary supplements’ [1]. However, certain situations (i.e. compromised energy intake, poor diet quality, low nutrient absorption) may warrant an athlete to consider a vitamin or mineral supplement to meet their daily needs, and although this is appropriate, given the potential harm of over-supplementing or consuming supplements of banned nature, the choice of approach taken in such instances should be determined in consultation with a nutrition expert. Accordingly, it is important to remember the thoughts of Larson-Meyer et al., [2], that ‘Supplement intake cannot reverse poor food choices and an inadequate diet’; notwithstanding, the words of Close et al., to adopt a ‘food first but not always food only’ approach [1] are also pertinent when it comes to replacing vitamins and minerals in athletes with compromised nutrient intake.

## Are Athletes at a Greater Risk of Deficiency?

There are a multitude of interacting factors that can contribute to sub-optimal nutrient status in athletes; these factors include processes such as increased excretion in sweat, urine and faeces, increased turnover, decreased absorption in the gastrointestinal tract, and biochemical adaptation to training [3, 4]. Given the high training demands and overall daily energy expenditure of elite athletes, greater exposure to the potential for exercise-induced vitamin and/or mineral deficiencies appears logical. However, broad vitamin and mineral deficiencies are uncommon in athletes [5], since overall energy intake generally increases to meet the energy requirement of the training demand, which affords appropriate replenishment of the greater micronutrient needs. Regardless, it is not always the case that energy intake increases commensurate with training load. For example, it is not uncommon for energy availability to be compromised in endurance athletes with heavy daily training schedules, where repeated bouts of exercise throughout the day can negatively impact the opportunity to adequately replenish energy requirements between sessions [6]. Further, the consumption of high-energy processed foods (i.e. sport gels, bars, and other highly processed products) in such athletes can lead to poor nutrient composition and quality within the diet, even if energy intake is appropriate. Finally, in weight sensitive or aesthetic sports, low energy availability is commonly reported due to restricted dietary intake in an attempt to manipulate body composition [7]. Such compromised or restricted nutrient intake not only impacts an athlete’s energy status, but also their opportunity to replace any vitamins and minerals utilised or excreted during exercise (i.e. B vitamins in energy production or electrolytes and iron in sweat) or those used in the recovery from/adaptation to the training stimulus (i.e. iron in red blood cell production) [8]. Notwithstanding, it is also likely that the inflammatory responses to heavy training may impact the ability of an athlete to absorb various nutrients at the level of the gut [9], which over time can also contribute to a greater exposure to risk of nutrient deficiency. For instance, exercise-induced increases in the inflammatory cytokine interleukin-6 have been linked to increases in the liver-produced peptide, hepcidin, which when elevated, functions to decrease iron absorption in the gut [10], a process recognised to be a contributor to the commonly seen compromised iron stores of athlete populations [11]. To this end, it is clear that athletes present with a greater overall need for the considered intake of vitamins and minerals, with an increased risk of deficiency likely relevant to individuals where energy intake and replenishment are poor and/or training load is high.

## What are the Assessment Frameworks to Explore a Deficiency?

Underlying nutrient deficiencies in athlete populations may impact a variety of health and performance outcomes. Commonly, symptoms of nutrient depletion in athlete populations tend to initially present as feelings of lethargy and fatigue [12], which may eventually impact training consistency, and therefore performance, over time. Given these potential negative implications for performance, it is integral that the nutrition support team working with athletes have a well-structured process of assessment to uncover nutrient issues prior to it affecting performance; this approach can also provide a comprehensive assessment tool to evaluate the need for dietary supplements when addressing any identified problems. In an attempt to provide this structure, a modified A–E framework of adapted nutritional assessment has been previously proposed [2, 13]. This framework provides a comprehensive assessment of nutritional status across five key domains, including:

- Anthropometrics, to assess an athlete’s body composition and any changes over time,
- Biochemical analysis, to assess the presence and levels of targeted biomarkers (e.g. iron, vitamin D [25(OH)D], etc.) in the blood, saliva and/or urine against commonly accepted thresholds,
- Clinical assessment, to provide insight into the presence of any relevant history and/or symptoms impacting the athlete,
- Dietary analysis of nutrient intake over time via various methods of food intake recall,
- Environment scan, entailing an assessment of the athlete’s surroundings to explore the impact of factors such as living setting, social and cultural factors, training programming, etc. on nutrient intake as an underlying cause of any potential issues.

It should be noted that individual aspects of this comprehensive assessment approach may be conducted by numerous individuals within the sports medicine team supporting an athlete (i.e. dietitian, sports physician, etc.), highlighting the multidisciplinary approach to athlete service provision. In combination, the interrogation of these five factors provides a comprehensive assessment tool to enable the detection of nutrient disorders, the potential contributing factors to the disorder, and the approach (i.e. nutritional correction or supplementation) to address any identified problem(s).

## What are the Strategies to Prevent and Treat Nutrient Deficiency?

If a nutrient deficiency is uncovered, consideration needs to be given to numerous underlying factors that could influence the success of any intervention. One significant factor might include the approach taken to correcting the issues. For instance, for any given identified nutrient deficiency, there are several approaches that can be taken to improve the situation. An initial approach might be a ‘food first’ prospect, whereby there is a concerted push to increase the availability of the lacking nutrient in the athlete’s overall daily energy intake [1, 2]. Although this food first approach is a preferred starting point for any nutritional intervention, the efficacy of this approach is only at its highest when there is an obvious and addressable issue with diet composition and/or energy intake. However, it is not always possible to correct a nutrient deficiency by simply advising the athlete to eat more, especially when a deficiency generally requires more of the specific nutrient to fix the problem than the typical Recommended Dietary Intake (RDI) [1]. Of note, RDI is the average amounts of specific nutrients required daily for sustenance or avoidance of deficiency states [14].

Given this issue, a concurrent approach to increasing nutrient density from food might be to explore the addition of a specific oral supplement (i.e. relevant to the observed nutrient deficiency) into the athlete’s daily nutrition routine (e.g. consuming a daily iron supplement to make up for low overall iron intake in a vegetarian diet). In general, the efficacy of this approach is usually high; however, the process to replete an issue is usually slow (i.e. at least 4–12 weeks in the case of vitamin D [15, 16] and iron [17, 18]), could result in some level of gastrointestinal (GI) distress, and as with any commercially available supplement, may be accompanied by some level of risk relevant to supplement contamination [19]. Therefore, a well-considered approach using batch-tested supplements should be undertaken.

Given the length of time required to correct a deficiency through food first and/or oral supplementation, a final approach available for some deficiencies (e.g. iron and B vitamins) might be to consider infusion of the depleted nutrient directly into the circulation (i.e. parenteral supplementation). Given that most nutrient deficiencies have their origin at the level of the gut, parenteral approaches to supplementation are attractive since they bypass this issue, and their effect is immediate. However, parenteral approaches to nutrient supplementation are associated with their own set of risks [20], such as adverse reactions to the infusion itself, issues and (well-justified) stigma with needle policies in elite sporting organisations [21] and antidoping rule considerations for routes of administration that must be considered. Accordingly, it is not uncommon that this final approach to nutrient supplementation is generally reserved for more severe and persistent cases of deficiency, and therefore should only be considered in collaboration with a trained sports physician.

Once the approach of supplementation is determined, other considerations for the practitioner working with athletes who present with an identified nutrient deficiency might include:

- The dose (which will likely be greater than the RDI to correct an issue) and formulation of the supplement,
- The timing of supplement consumption (i.e. time of day and proximity to exercise or feeding),
- The interactions of the supplement with other food (e.g. co-consumption of iron and calcium can decrease the absorption of iron, whereas co-consumption of iron and vitamin C can enhance the effect [22]),
- Any food–drug interactions that might be of relevance (e.g. oral contraceptives negatively impacting folate metabolism, etc. [2]),
- The duration of supplement consumption (i.e., how many weeks will be required to see a positive impact and return to healthy levels),
- The frequency of biochemical monitoring to assess efficacy of effect.

Clearly, there are a myriad of factors to consider when determining the best approach to addressing an athlete’s nutrient deficiency, with specific nuances relevant to the lacking nutrient likely needing attention when devising the best way to rectify the issue.

## Important Vitamins and Minerals for Athletes

When considering the numerous vitamins and minerals that exist in our diet, and the innumerable functions they play, it becomes clear that a comprehensive review of them all would be impractical (although readers are referred to Beck et al. [23] for further detailed review). However, from an athlete-centric perspective, there are some key vitamins and minerals that are integral to adaptation and optimal function. Accordingly, we have delimited our attention here to several key nutrients (Table 1) of importance to haematology, bone health and immune function, as key (and common) contributors to an athlete’s potential for adaptation, and their overall health.

**Table 1:** Key vitamins and minerals for athletes of importance to haematology, bone health and immune function, their Recommended Daily Intake (RDI) by sex, the blood reference range for healthy adults (18–50 years) and their common food sources

	RDI^a^		Blood reference range^b^	Common food sources
	Male	Female		
Vitamin				
Vitamin C	90 mg	75 mg	4–14 mg/L	Citrus (oranges, kiwi, lemon, grapefruit), bell peppers, cruciferous vegetables (broccoli, Brussels sprouts)
Vitamin D	15 µg	15 µg	> 50 nmol/L	Salmon, tuna fish, sardines, beef liver, egg yolk, milk
Vitamin B12	2.4 µg	2.4 µg	140–1000 pmol/L	Fish, liver, red meat, eggs, poultry, milk, cheese, yoghurt
Vitamin B9 (folate)	400 µg	400 µg	> 7 nmol/L	Leafy green vegetables (spinach, asparagus, Brussels sprouts), beans, peanuts, liver, fish, eggs
Minerals				
Iron	8 mg	18 mg	30–300 µg/L (ferritin)	Red meat, chicken, leafy green vegetables (spinach, bok choy), nuts, seeds
Calcium	1000 mg	1000 mg	210–260 mmol/L	Milk, cheese, yoghurt, soybeans, leafy green vegetables (spinach, bok choy), almonds
Zinc	14 mg	8 mg	9–16 µmol/L	Shellfish, beef, chicken, legumes, nuts, seeds

^a^Nutrient reference values are based on the National Health and Medical Research Council (Australia) recommendations [14]

^b^Blood reference ranges, https://pathwesttd.health.wa.gov.au/testdirectory/

### Haematology

From a haematological perspective, iron, folic acid and vitamin B12 are significant contributors to red cell production [24]. The importance of iron is well established for its critical role in the formation of haemoglobin incorporated within red blood cells and enzymes integral to the electron transport chain at a cellular level. Consequently, iron is integral to key processes including oxygen transport and energy production, which are highly relevant to athletes [25]. However, iron deficiency is reported to affect ~ 10% of male and ~ 35% of female athletes [26] and is among the most common nutrient disorders reported in athlete populations. Currently, it is established that there are three stages of iron deficiency, which progressively increase in symptoms and severity of effect as the depletion of serum ferritin (sFer), haemoglobin (Hb) and transferrin saturation (TSAT) progress [27]. Stage 1 is iron depletion (ID), characterised by a reduction in sFer without impact on red blood cell production. Stage 2 is iron deficiency non-anaemia (IDNA) which presents with further depleted sFer, causing erythropoiesis to diminish as the iron supply to the erythroid marrow is reduced (as evident by a decrease in TSAT). Stage 3 is iron-deficient anaemia (IDA) which represents the most serious and debilitating level of compromise, where low sFer and TSAT have progressed to a decrement in Hb concentration. Here, not only do athletes report the common feelings of lethargy and fatigue (as they do in stage 1, iron depletion), but they also present with reductions in overall physical capacity [11]. Previous work has developed athlete-specific blood screening [11] and iron supplementation frameworks [22], thus providing practitioners with guidelines and strategies to mitigate the progression of stage 1 iron depletion to a more severe stage of impact (i.e. stages 2 and 3). Interestingly, current data suggest that iron supplementation in the absence of severe deficiency (i.e. IDA) is unlikely to result in performance benefits for athletes, with meta-analyses demonstrating unchanged performance outcomes in iron-deficient non-anaemic (IDNA) athletes [28]. However, iron supplementation provided to IDNA individuals has been shown to improve haemoglobin and ferritin concentrations, whilst also reducing the subjective feelings of fatigue during exercise [29], which can impact the quality and consistency of training over time. Furthermore, under exceptional circumstances of environmental stress, such as altitude training designed to stimulate red cell production, iron supplements should be considered in athletes with suboptimal ferritin stores, in an effort to meet the additional erythropoietic demands of the hypoxic stimulus [11, 30].

In addition to iron, it is also well recognised that B vitamins have an important role in haematological function in active populations [31]. Of the nine B vitamins found within the diet, folate (B9) and cobalamin (B12) play crucial roles in facilitating the production of red cells in the bone marrow [32]. Interestingly, clinical B12 [33] or folate deficiency [34] can result in megaloblastic anaemia, due to disruption of DNA synthesis and repair that results in ineffective erythropoiesis [24]. Notably, pernicious anaemia, a form of megaloblastic anaemia, occurs due to B12 deficiency and can only be treated with parenteral administration of B12 due to a lack of gastric intrinsic factors required for B12 absorption [35]. In athlete populations, observational data suggest that low circulating levels of B12 are mildly associated with lower haemoglobin concentration and haematocrit, and that B12 supplements may be beneficial to haematological adaptation when suboptimal levels are detected in the blood [36]. However, standardised thresholds for the classification of B12 deficiency in athletes are not well defined, and therefore, further work is required to establish best practice guidelines for addressing this issue. Of note, the impact of low B12 levels is especially pertinent to vegetarian and vegan athletes, since B12 is found more readily in animal food sources. Accordingly, athletes adhering to certain dietary restrictions may need to be mindful of relevant sources of B12 in their diet, and not averse to biochemical and clinical assessment of B12 and iron stores if persistent feelings of lethargy are present.

### Bone Metabolism

When considering bone health, vitamin D [37] and calcium [38] have been extensively studied in athlete populations. Vitamin D is known to play an important role in calcium homeostasis, which is essential for bone health, thus having a positive effect on mitigating fracture risk. Vitamin D can have a positive effect on osteoblasts and bone remodelling via induction of receptor activator of nuclear factor-κB ligand (RANK-L) and phosphate homeostasis [39]. Collectively, such factors, in combination with the mechanical loads of exercise, are hypothesised to stimulate mitogen-activated protein kinase signalling, which may promote increased bone mineral density and lower fracture risk (see [37] for review).

Current literature indicates that any benefits of vitamin D supplements on athlete health likely depend on circulating levels of 25-hydroxycholecalciferol (25[OH]D), with between 30% and 39% of elite athletes being reported to present with insufficiency (< 50 nmol/L) [40]. Importantly, there appears to be no ergogenic benefit of providing doses of supplemental vitamin D to elevate 25[OH]D beyond sufficiency (> 75 nmol/L), with excessive supplementation using mega dosages resulting in excessive vitamin D levels (e.g. 25[OH]D > 180 nmol/L), which may ultimately be detrimental to one’s health [37]. Such events are often accompanied by severe hypercalcemia and hypercalciuria, as well as low parathyroid hormone activity, which may compromise bone integrity [41]. Interestingly, meta-analyses of male and female athletes and military personnel (participating in a variety of activities such as soccer, baseball, dancing, swimming, American Football and military recruit training) report that low 25[OH]D (< 30 ng/mL) is associated with greater risk for stress fractures (*n* = 7 studies, 3625 participants) [42], highlighting the importance of maintaining adequate 25[OH]D levels and regular screening regimes.

In addition to vitamin D, low serum calcium caused by dietary insufficiencies is known to stimulate an increase in parathyroid hormone (PTH) and osteoclast activity, inducing a catabolic effect on bone [43]. Importantly, habitual low dietary calcium intake is associated with poor bone mineral density [44], with purported causes suggested to include insufficient/restrictive energy intake, the avoidance of certain foods (i.e. dairy products) and/or lactose intolerance [8]. Such scenarios may benefit from calcium supplements to support bone health. Finally, a reduction in serum ionised calcium occurs during exercise, prompting an increase in PTH activity and bone resorption [45]. Interestingly, pre-exercise calcium intake (1000 mg) has been shown to minimise perturbations of bone calcium homeostasis [46], and therefore, this approach might be considered for athletes at heightened risk of bone injury. In athlete populations, a calcium RDI of 1500 mg/day has been suggested appropriate to meet increased metabolic demands and support bone health [47]. To maximise absorption, this calcium intake should be apportioned in smaller doses (i.e. 500 mg portions) throughout the day [48], which can be found in food sources such as dairy products (e.g. milk, yoghurts, cheese) or plant-based foods (e.g.,green leafy vegetables, broccoli, soybeans, fortified plant-based milks).

### Immune Function

Sub-optimal nutrition is a major risk factor for illness and infection in athletes [49], with low energy availability often highlighted as a major consideration [7]. A range of nutrients are known to play a significant role in immune function (i.e. iron, vitamins A, D, E, B6, B12; for review see [50]). For instance, vitamin D is reported to play an important role in both innate and acquired immunity, with numerous reports presenting a case for an inverse relationship between vitamin D concentrations and upper respiratory infection (URI) in athletes and military personnel (for review see [37]).

In addition to vitamin D, zinc and vitamin C are commonly considered supplements to help improve immune function [51]. Zinc is reported to play an important role in nucleotide and nucleic acid synthesis, whilst also acting as an antiviral agent by increasing interferon gamma, thus decreasing the docking of common cold viruses with binding sites [52]. When considering URI, the potential antioxidant and anti-inflammatory influence of zinc may also contribute towards the tolerogenic effects on immunity, with recent meta-analyses reporting that the duration of URI was reduced by 33% (translating to ~ 3 days) if 75 mg/day of elemental zinc was provided within the first 24 h of illness symptoms developing [53]. Interestingly, a previous systematic review has reported that athletes often present with lower circulating zinc concentrations than the general population, despite their greater dietary intake [54]. This suggests that the zinc requirements of athletes are likely higher than the general population, possibly due to sweat losses experienced as part of extended periods of intense training [55].

Often promoted for its antioxidant capacity [56], vitamin C is an active scavenger of reactive oxygen species in intra- and extra-cellular fluid [57]. During infection, vitamin C levels in leucocytes can fall by up to 50% [58], which have been linked to increased oxidative stress. Such events provide rationale for vitamin C intake when attempting to preserve athlete health, especially during infection or during heavy training where inflammation and oxidative stress are acutely increased. In fact, meta-analyses of 18 randomised controlled trials in exercising populations (conducted from 1998 to 2019; *n* = 313 participants) showed vitamin C supplementation attenuated the oxidative stress and inflammatory response to a single bout of exercise [59]. Interestingly, clinical trials have reported a dose–response relationship between vitamin C supplementation and URI duration, with large daily oral dosages between 3 to 8 g [60, 61]. Furthermore, a 2013 Cochrane review reported that in five studies conducted from 1961 to 1996, low-dose vitamin C supplements (0.25–1.0 g/day) provided to ‘heavy exercisers’ (*n* = 589 marathoners, skiers and military personnel) reduced the incidence of URI by ~ 52% [62]. Such findings have led to the common suggestion that vitamin C supplements might be considered for athletes during periods of heightened infection risk, such as extended periods of heavy training or travelling for key competitions [63].

## Special Considerations for Female Athletes

Females have distinct biological and phenotypical attributes which make the nutrient needs of female athletes unique. In females, the sex hormones, oestrogen and progesterone, play an important role in reproductive development and menstruation [64], signalling the release of other hormones (such as luteinizing hormone and follicle stimulating hormone), which also have important roles in ovulation and maintaining pregnancy. Female ovarian hormones not only change across the life span (from puberty through to menopause), but also change cyclically throughout the four phases of the menstrual cycle (1—menstruation phase, 2—follicular phase, 3—ovulation phase, 4—luteal phase). For instance, during menstruation, concentrations of both ovarian hormones are low [65]; however, oestrogen levels increase during the follicular phase to stimulate ovulation, prior to high circulating concentrations of both oestrogen and progesterone during the luteal phase. Of note, concentrations of these hormones may also change due to other stimuli, such as pregnancy, hormonal contraception use or diet/exercise interactions [65]. In addition to their reproductive roles, ovarian steroids can influence a variety of physiological and biological processes, such as thermoregulation, metabolism, cognition and autonomic regulation [65–67]. When considered collectively, the implications of these physiological alterations may extend to impact athletic performance. Indeed, a recent meta-analysis concluded that exercise performance might be trivially reduced during the early follicular phase of the menstrual cycle [68]; however, there were numerous issues with the available data, such that the majority of studies were considered to be of ‘low’ quality, and there was large between-study variance identified, making it difficult to infer any practical conclusions for elite female athletes. Regardless, it is evident that female athletes more commonly present with certain micronutrient deficiencies [69, 70], and therefore, nutritional approaches that consider the unique needs of female athletes are required.

Currently, there is limited research exploring the periodisation of nutrition around the menstrual cycle. However, there are minerals which merit closer investigation, such as the intake of iron. This is particularly important to menstruating female athletes who might experience losses equating to between 5 and 40 mg of iron each cycle [71, 72]. Of note, this number is likely much higher in women with heavy menstrual bleeding (HMB), a condition thought to be highly prevalent in athletic cohorts [73]. Interestingly, hepcidin, the aforementioned iron regulatory hormone responsible for controlling iron absorption, has been shown to be downregulated by oestrogen [74], which means that iron absorption might be increased when oestrogen levels are high (i.e. follicular phase of the menstrual cycle). This may provide a window of opportunity for females to recover the net iron loss from menstruation. While mechanistic support for this association is strong, studies of human trials are unclear [75, 76] and no study has yet directly assessed changes in iron absorption (rather than hormonal alterations) across the menstrual cycle in female athletes.

Regardless, these menstrual blood losses and hormonal fluctuations are unique to female athletes, and result in a greater iron requirement compared with males. Indeed, this is reflected in the RDI guidelines for the general population, where iron consumption recommendations in females are more than double that of males (18 versus 8 mg/day) [14]. However, this RDI can be quite difficult for females to achieve as their generally smaller body size means a lower absolute energy intake is required when compared with males. The typical western diet provides ~ 6 mg of iron in every 1000 kcal [77]; therefore, females are required to eat more nutrient dense foods to achieve their dietary iron target within their caloric requirements. To compound this issue, female athletes commonly follow diets that are restrictive, including vegetarian or vegan diets low in quality (haem) iron sources [11], or those limiting either carbohydrate or energy intake [78], which have been shown to contain lower amounts of dietary iron. Accordingly, inadequate energy intake, and therefore lower dietary iron intake, contributes to the higher incidence of iron deficiency seen in female athletic populations [79]. Consequently, an increased focus on either dietary or supplemental iron intake in female athletes is required, particularly where additional challenges to iron balance occur from high exercise loads. This is reflected in the iron screening recommendations for athletes [11], which suggest that females should have their iron status assessed at 6-month intervals, especially in cases of known iron compromise. The more frequent routine screening of women allows minor deficiencies in iron (i.e. stage 1 iron depletion) to be treated prior to progression towards severe stages of the nutrient disorder (i.e. stage 3 IDA).

In addition to iron, folate is a B vitamin of particular importance to pre-conception and throughout pregnancy. As described previously, both iron and folate have fundamental roles in erythropoiesis [80]. Further, folate coenzymes are essential in nucleic acid synthesis, methionine regeneration, and in one-carbon metabolism [81], making them particularly important during periods of rapid growth (i.e. during pregnancy). Female athletes training throughout their pregnancy should strongly consider both iron and folate supplementation, as the added haematological stress of high exercise volumes on top of those associated with foetal growth and development may accelerate the progression of a deficiency, if not treated proactively. Indeed, during pregnancy, the RDI for dietary iron increases from 18 to 27 mg/day [14] to accommodate the ~ 2-fold increase in blood volume [82]. Similarly, folate requirements are estimated to be ~ 2-fold higher [83], as reflected in the recommended daily allowances, which increase from 400 to 600 µg dietary folate equivalents (DFE). These recommendations are based on the general population, as athletic specific values do not currently exist; however, they should be generally applicable assuming a pre-existing deficiency is not evident. Nevertheless, given the lack of research in the area, individualised consultation and screening for nutrient deficiencies throughout pregnancy is strongly advised. Interestingly, there is evidence to suggest that oral contraceptive use is also associated with a reduction in plasma folate concentrations and red blood cell folate concentrations [84]. Accordingly, athletes using oral contraceptives may need to consider higher folate consumption, particularly if planning pregnancy. Of note, many prenatal vitamins will contain between 800 and 1000 µg DFE of folic acid, which will support reproductive health and provide protective effects on the foetus when used for 3–6 months prior to conception [85].

Clear associations between menstrual function, bone health and energy availability have previously been described via the Female Athlete Triad [86] and more recently as part of the Relative Energy Deficiency in Sport (REDs) model [87]. Female athletes with amenorrhoea often present with poor bone mineral density, bone microarchitecture and/or bone turnover markers, placing them at an increased risk of stress fractures [88, 89]. Considering that peak bone mass is often attained by 30 years of age, and that 95% of a young woman’s peak bone mass is present by age 20 [90], this would substantially increase the risk of developing early onset osteoporosis, which will present as a major complication in late-life. Interestingly, in a single study of male and female athletes, low energy availability [(LEA) 15 kcal·kg·FFM^−1^] resulted in negative alterations to bone turnover markers (BTMs) following exercise in only female participants [91].While acknowledging the limitations associated with using BTMs to infer bone health, it may allude to an ability for men to better tolerate caloric restriction compared with women [92]. While vitamin and mineral intake can help support bone health, it is less effective when low energy availability is present, and in these instances optimising energy intake should be the primary nutritional intervention to improve poor bone health.

Overall, when considering the unique challenges of female athletes and the associated needs for vitamin and mineral intake, it should be mentioned that a recent audit on the representation of women in research examining iron, calcium and vitamin D supplementation [93] found not one study was able to classify and report menstrual status according to best practice guidelines [65]. Furthermore, only 13% of all study participants in this research could be classified as female athletes [93]. Therefore, without research effectively reporting and controlling for menstrual status, or even using female athletic populations, it is not currently possible to provide conclusive recommendations for vitamin and mineral intake specific to an athlete’s menstrual status or menstrual cycle. Accordingly, a greater effort to explore the unique nutrient needs of the female athlete is warranted.

## Summary and Conclusions

Athletes are regularly exposed to high levels of training stress, and therefore, their energy intake needs to match the energy demand. However, there are a variety of factors that contribute to the poor replenishment of energy needs in athletes, and as such, the replenishment of key vitamins and minerals can be compromised, putting athletes at an increased risk of nutrient deficiencies. Given this increased risk, it is important that practitioners use a robust framework to assess the overall energy requirements, the current dietary practices and the biological and clinical status of their athletes, to identify if and when an athlete may require nutritional intervention. Should a nutrient deficiency be detected, it is important to consider the appropriate approach to correcting the problem, whilst also accounting for the various factors (e.g. the nutrient RDI, supplement dose/timing, co-consumption of other foods and any food–drug interactions) that may impact the efficacy of the approach. Of note, there are numerous vitamins and minerals of key importance to athletes, each having specific relevance to certain situations (e.g. iron and B vitamins are significant contributors to haematological adaptation). Accordingly, the usefulness of supplementation for a given situation should be carefully considered before use, and supplements should be consumed to augment an athlete’s typical diet, remembering the adage that ‘supplement intake cannot reverse poor food choices and an inadequate diet’ [2]. Finally, it should be noted that the female athlete has nuanced vitamin and mineral requirements that differ to their male counterparts, and that the research landscape requires significant work to better understand the unique nutritional challenges they face.
